# Utilization of Primary Healthcare Centers by Residents of Ido-Ekiti, Nigeria

**DOI:** 10.4314/ejhs.v33i2.7

**Published:** 2023-03

**Authors:** Tunrayo Oluwadare, Oluwaseun Adegbilero-Iwari, Ayodeji Fasoro, Charles Faeji

**Affiliations:** 1 Department of Community Medicine, Afe Babalola University, Ado- Ekiti, Nigeria; 2 Department of Public Health, Afe Babalola University, Ado- Ekiti, Nigeria; 3 Faculty of Health, University of Canterbury, Christchurch, New Zealand; 4 Department of Medical Microbiology and Parasitology, Afe Babalola University, Ado-Ekiti, Nigeria

**Keywords:** Primary health care, rural area, health systems, utilization, Nigeria

## Abstract

**Background:**

Primary health care (PHC) centers help in providing a complete, universal, unbiased, and reasonable healthcare service to all. One major aim of PHC is to reduce health inequality. Most PHC centers in Nigeria cannot deliver fundamental healthcare services due to staffing, equipment distribution, quality infrastructure, and drug supply problems. The objective of this study was to assess the awareness and utilization of PHC services in a rural community in Nigeria.

**Methods:**

The study was carried out in a pastoral area in Ekiti State, Nigeria. A multistage sampling procedure was used to recruit adults aged 18 years and over residing in 361 households. A semi-structured questionnaire was utilized for data compilation. Study data were evaluated using IBM SPSS version 28.0 and reported using descriptive and inferential statistics. Chi-square test and binary logistic regression were used to assess the associated factors and predictors of PHC utilization.

**Results:**

The proportion of those who had ever utilized PHC services was 45.7%. Significant predictors of the utilization of PHC centers include knowledge of the location of a PHC center, awareness that PHC centers operate 24 hours every day, and awareness that community members are part of the PHC staff.

**Conclusions:**

Non-availability of medical personnel and ease of access to secondary and tertiary health institutions are potential threats to the use of PHC facilities.

## Introduction

Primary health care (PHC) is defined as “critical health care founded on practical, systematically sound, and generally acceptable methods and technologies made universally available to individuals and families in the community through their full involvement and at a price that they can afford to preserve every stage of their development in the essence of self-dependence and self-purpose” (1). The Declaration of Alma-Ata formally adopted primary health care (PHC) to provide a complete, universal, impartial and reasonable healthcare service to everyone (2). This was aimed at addressing health inequalities that exist all over the world. In Nigeria, the pioneer comprehensive federal health policy based on PHC was launched in 1988 (3). The PHC program prioritized “health for everyone” as the key strategy for implementing the policy at all federal, state, and local governments supporting a three-tier healthcare system involving multi-sectoral inputs, community involvement, and non-governmental health providers (4). The local government manages the PHC, which serves as the initial point of access for persons. The state controls secondary health care, which allows referrals from PHC clinics and provides services further than primary capability while tertiary care is assisted by advanced diagnostics and current technology. The federal government oversees tertiary care centers (5).

There are only about 20% of Nigeria's 30,000 PHC centers operational (5). Most PHC centers in Nigeria cannot deliver fundamental healthcare services due to staffing, equipment distribution, quality infrastructure, and drug supply concerns (6–8). The impact of these on health status and PHC use cannot be overemphasized. PHC facilities' incapacity to deliver basic medical services to Nigerians has led to an inflow of patients in secondary and tertiary care (6). Health is a social right, and governments should prioritize it (9). Policymakers in Nigeria do not prioritize healthcare despite sufficient evidence linking health and economic development. Decision-makers have struggled to increase health spending (10, 11). A breakdown of Nigeria's 2022 budget indicated that only 4.2% was allocated for healthcare across the 36 states of the federation and the Federal Capital Territory (12). This means that an average Nigerian is only entitled to 3,510 Naira (US$ 8.37) worth of medical care in 2022. The 4.2% allocation falls short of the 15% annual budget pledged by heads of state of African Union countries in April 2001 at the Abuja Declaration (13).

Less than half of Nigeria's rural population have access to modern healthcare (10, 14) and this seems not to be improving as these rural areas' involvement in making logical health promotion decisions is also limited (15). Other issues include a dearth of basic health data for the Nigerian general population, poor fiscal resource allotment to health services, particularly, in high-priority locations, flawed basic groundwork, and inadequate logistical assistance (5, 6, 16).

PHC is based on the following basic elements: health education, appropriate nutrition, family planning, vaccination, prevention of endemic illnesses, satisfactory water supply and basic cleanliness, appropriate treatment of common diseases, essential medications, community mental health, oral health, and referral system (7). Many of these are lacking in impoverished nations, particularly in Africa and as a result, individuals have poor knowledge of their national health programs and do not take advantage of PHC centers effectively. Also, socioeconomic, psychological, demographic, and geographical constraints affect their access to PHC services (15). Nigerians have the right to quality health care just as everyone else in developed countries. Literature investigating the utilization of PHC services in Ekiti State especially, Ido-Ekiti, Nigeria remains sparse. This study sought to assess the awareness and use of PHC centers in a rural community in Nigeria.

## Methods

**Study area and design:** The study was carried out in Ido-Ekiti, which is in Ido-Osi local government area of Ekiti State, Nigeria. Ido-Ekiti had an estimated population size of 239,600 in 2022 (17). Ido-Ekiti is majorly dominated by the Yorubas and has 10 settlements. It is bordered by Orin-Ekiti, Usi-Ekiti, Ilogbo-Ekiti, Ora-Ekiti, and Igbole-Ekiti. Despite being a rural area, the town has a Federal Teaching Hospital, three PHC centers, and one private-owned clinic. A community-based cross-sectional design was adopted to investigate the utilization of PHC centers by residents of Ido-Ekiti. The study population was residents of Ido-Ekiti, and all consenting adults aged 18 years and over residing in Ido-Ekiti were included in this study. Those with intellectual disabilities and who could not give consent were excluded from this study.

**Sample size determination**: The sample size needed for the study was calculated using Fisher's formula for population size>10,000. The sample size was adjusted for potential non-respondents and was calculated as 10% of the total sample size. The minimum sample size needed was 370 participants. Sampling with probability proportionate to size (PPS) was used to establish the number of respondents needed from each settlement.

**Sampling technique**: A multistage sampling technique using the probability proportionate to size (PPS) approach was used to select the participants in this study. At the first stage, four out of 10 settlements in the town were selected using simple random sampling of balloting. The settlements selected are Iyedi, Alapo, Oke Bareke, and Isolo. At the final stage, households in each settlement were selected systematically by using a sampling interval. The sampling interval was determined by dividing the estimated number of households in the selected settlements by the estimated sample size needed. The oldest adult available in each household at the time of the visit who met the inclusion criteria was recruited.

**Study instrument and data collection**: The instrument used for data collection was a semi-structured questionnaire. It was designed after reviewing past literature on PHC utilization. The study instrument was divided into three parts: Section A — Socio-demographic characteristics of the respondents, Section B — Awareness of respondents on PHC centers, and Section C - Utilization of PHC centers. The face and content validity were ascertained by experts in the department of Public Health, Afe Babalola University Ado-Ekiti. The reliability of the items in the questionnaire was assessed using Kappa statistics. The questionnaire was administered to 20 households in one of the six settlements that was not included in the study. The questionnaire was re-administered to the same respondents 20 days later. The Kappa statistics show the proportion of agreement ranged from 0.7 — 1.0. Community advocacy was done before data collection. The purpose and benefits of the research were clarified to the community members. The questionnaire was administered by Afe Babalola University undergraduate medical students. The medical students were community residents and were undergoing medical training at Federal Teaching Hospital Ido-Ekiti. Data collection was done from March-May 2021. Households in the four settlements were selected systematically using a sampling interval, and the oldest adult who met the inclusion criteria at the time of the visit was interviewed. The questionnaire was interviewer-administered. Utilization of PHC was assessed by asking if the respondents had ever received any form of treatment from the PHC center(s) in the study location.

**Data analysis**: Data were checked for errors, cleaned, and manually entered for analysis. IBM SPSS version 28.0 was used. Data were analyzed using both descriptive and inferential statistics. Descriptive statistics were presented in frequencies, percentages, means, and standard deviation. The inferential procedure involved the use of Pearson's Chi-square and logistic regression. Pearson's Chi-square test was used to assess if there was a relationship between categorical variables. Multivariable analysis using a binary logistic regression model was used to determine factors that significantly predict the utilization of PHC services. Fourteen significant factors when the p value was set at 0.25 in the bivariate analysis were included in the final regression model. This p value is recommended because the use of the traditional level (p<0.05) often fails to identify some variables that are known to be of importance (18).

**Ethics**: The confidentiality of participants' responses and the anonymity of their identities were fully guaranteed. Ethics approval to conduct the study was sought and obtained from Afe Babalola University Ethics Committee and Federal Teaching Hospital Ethics Committee, Ido-Ekiti before the respondents were approached. This is akin to an Institutional Review Board (IRB) approval. All procedures performed were in accordance with the ethical standards of the institutional and/or national research committee on medical research involving human participants and with the 1964 Helsinki Declaration and its later amendments.

## Results

Out of the 370 households approached, complete data were obtained from 361 respondents. This study revealed that the respondents' mean age was 32.25 ±10.98 years and the median age was 30 years. Table 1 shows that the majority of the respondents were females (60.7%), aged less than 40 (78.2%), married (47.9%), had tertiary education (51.3%), and Christians (86.4%).

**Table 1:** Socio-demographic characteristics of respondents (n=361)

Variable	Frequency	Percent
**Age (Years)**		
< 30	168	46.6
30–39	114	31.6
40–49	46	12.7
50 & above	33	9.1
Mean (SD)	32.3 (11.0)	
**Gender**		
Male	142	39.3
Female	219	60.7
**Marital status**		
Married	173	47.9
Never married	167	46.3
Separated	13	3.6
Widowed	8	2.2
**Educational qualification**		
No formal education	18	5.0
Primary	20	5.5
Secondary	138	38.2
Tertiary	185	51.3
**Ethnic group**		
Yoruba	304	84.2
Igbo	41	11.4
Hausa	8	2.2
Others	8	2.2
**Occupation**		
Civil servant	93	25.8
Farmer	21	5.8
Self-employed	93	25.8
Trader	67	18.6
Artisan	25	6.9
Unemployed	62	17.1
**Religion**		
Christianity	312	86.4
Islam	40	11.1
Traditional	5	1.4
Others	4	1.1
**Estimated income per month (Nigerian Naira)**		
<10,000	55	15.2
10,000 – 49,999	172	47.7
50,000 – 99,999	83	23.0
≥100,000	51	14.1

1 US dollar = 419.39 Naira

The respondents' awareness and utilization of the service components provided by PHC centers revealed that the majorities were aware of immunization services (82.8%), treatment of common diseases and injuries (78.9%), and antenatal care services (72.3%). However, less than one-fifth of the respondents utilized each of these services (Table 2). Table 3a shows that there was no association between socio-demographic variables and utilization of PHC, although 45.7% of the respondents had ever utilized a PHC center in the study location. Nevertheless, a significant association (p<0.05) was observed between the utilization of PHC and the knowledge of any PHC center in the study location, the knowledge of the provision of free services by the PHC centers, the knowledge that the PHC centers run for 24 hours, the knowledge of the existence of a Community Health Committee, and the knowledge of community members being part of the working staff at the PHC centers (Table 3b). Topmost among the reasons cited for the non-utilization of PHC centers were preference for General/Teaching hospitals (26.3), limited services provided by the PHC centers (23.3%), and shortage of supplies/drugs (20.8%) (Table 4).

**Table 2:** Respondents' awareness and utilization of service components provided by primary health care centers

	Awareness (Yes)		Utilization (Yes)	
Services	n	%	n	%
Immunization	299	82.8	51	14.1
Maternal and child health care	267	74	17	4.7
Family planning	241	66.8	26	7.2
Antenatal care services	261	72.3	18	5.0
Treatment of common diseases and injury	285	78.9	61	16.9
Health education	238	65.9	21	5.8
Provision of essential drugs	236	65.4	31	8.6
Prevention and control of locally endemic diseases	193	53.5	14	3.9
Promotion of proper nutrition	201	55.7	22	6.1
Promotion of basic sanitation	216	59.8	22	6.1
Dental and oral health	142	39.3	15	4.2
Mental health services	118	32.7	12	3.3
Referral services	208	57.6	21	5.8

**Table 3a:** Association between respondents' socio-demographic characteristics and utilization of primary healthcare centers

	Utilized PHC			

Socio-demographics	Yes	No	*χ* ^2^	*P*-value
	n(%)	n(%)		
**Overall**	165(45.7)	196(54.3)		
**Age (Years)**				
< 30	76(45.2)	92(54.8)	0.538	0.764
30–39	55(48.2)	59(51.8)		
40 & above	34(43.0)	45(57.0)		
**Gender**				
Male	58(40.8)	84(59.2)	2.229^y^	0.160
Female	107(48.9)	196(51.1)		
**Marital status**				
Married	76(43.9)	97(56.1)	0.296^y^	0.586
Not married	89(47.3)	99(52.7)		
**Educational qualification**				
No formal education	10(55.6)	8(44.4)	2.977	0.395
Primary	9(45.0)	11(55.0)		
Secondary	69(50.0)	69(50.0)		
Tertiary	77(41.6)	108(58.4)		
**Ethnic group**				
Yoruba	139(45.7)	165(54.3)	0.000^y^	1.000
Others	26(45.6)	31(54.4)		
**Employment status**				
Unemployed	27(43.5)	35(56.5)	1.665	0.435
Self-employed	100(48.5)	106(51.5)		
Paid employment	38(40.9)	55(59.1)		
**Religion**				
Christianity	142(45.5)	170(54.5)	3.073	0.380
Islam	18(45.0)	22(55.0)		
Traditional	4(80.0)	1(20.0)		
Others	1(25.0)	3(75.0)		
**Estimated income per month (Nigerian Naira)**				
<10,000	31(56.4)	24(43.6)	7.283	0.063
10,000 – 49,999	72(41.9)	100(58.1)		
50,000 – 99,999	33(39.8)	50(60.2)		
≥100,000	29(56.9)	22(43.1)		

*χ*^2^: Chi-square test

y Yates correction; Others: Ethnic and religious groups not defined.

**Table 3b:** Association between respondents' awareness and utilization of primary healthcare centers

	Utilization of PHC			
Awareness	Yes	No	*χ* ^2^	*P*-value
	n(%)	n(%)		
**Knowledge of any PHC center in the study location**				
Yes	156(54.7)	129(45.3)	44.487	**<0.001** *
No	9(11.8)	67(88.2)		
**Services provided are free**				
Yes	17(34.7)	32(65.3)	2.281^y^	0.131
No	148(47.4)	164(52.6)		
**Centers run for 24 hours**				
Yes	90(59.6)	61(40.4)	19.248^y^	**<0.001** *
No	75(35.7)	135(64.3)		
**A Community Health Committee exists**				
Yes	74(52.9)	66(47.1)	4.253^y^	**0.039** *
No	91(41.2)	130(58.8)		
**Members of the community are part of the working staff in the PHC center**				
Yes	77(59.7)	52(40.3)	14.952^y^	**<0.001** *
No	88(37.9)	144(62.1)		
**Aware of services provided at PHC centers**				
No	21(50)	21(50)	0.353	0.622
Yes	144(45.1)	175(54.9)		

*χ*^2^: Chi-square test

y Yates correction

* *p*-value < 0.05

**Table 4:** Reasons for non-utilization of Primary Healthcare centers

	Yes	
Reason	Frequency	Percent
Limited services provided by the PHC center(s)	84	23.3
Unsanitary condition of the center(s)	55	15.2
Non-availability of qualified medical personnel especially doctors	71	19.7
Attitude of health personnel	55	15.2
Shortage of supplies/ drugs in the health facility	75	20.8
Distance to the health facility	48	13.3
Cost of services	44	12.2
I prefer general/teaching hospitals	95	26.3
I prefer self-medication	38	10.5
I prefer traditional practitioners	26	7.2
No particular reason	40	11.1

Table 5 shows the result of the multivariable analysis. Significant predictors of the utilization of PHC centers include the knowledge of the location of a PHC center (AOR = 4.28, 95%CI = 1.86 - 9.84), awareness that the PHC operates for 24 hours every day (AOR = 1.66, 95%CI = 1.17 - 2.35), and awareness that community members are part of the PHC staff (AOR = 1.44, 95%CI = 1.04 - 1.99).

**Table 5:** Predictors of utilization of primary healthcare centers

Factor	B	p-value	AOR	95% CI	
				Lower	Upper
Knowledge of any PHC center in the study location	1.454	**0.001** *	4.281	1.862	9.843
Awareness of if services provided are free	-0.272	0.287	0.762	0.462	1.257
Awareness of if centers run for 24 hours	0.507	**0.004** *	1.661	1.172	2.353
Awareness of if a Community Health Committee exist	0.017	0.949	1.017	0.598	1.732
Awareness of if members of the community are part of the staff at the PHC centre	0.366	**0.026** *	1.442	1.044	1.991
Limited services provided by the PHC center	0.202	0.724	1.224	0.399	3.75
Unsanitary condition of the center(s)	0.083	0.882	1.086	0.364	3.243
Non-availability of qualified medical personnel especially doctors	-1.759	**0.004** *	0.172	0.051	0.578
Attitude of health personnel	-0.626	0.23	0.535	0.192	1.486
Shortage of supplies/ drugs in the health facility	0.861	0.132	2.365	0.771	7.255
Distance to the health facility	0.608	0.258	1.837	0.641	5.262
I prefer general/teaching hospitals	-1.967	**<0.001** *	0.14	0.062	0.313
I prefer self-medication	-0.301	0.543	0.74	0.281	1.952
No particular reason	-0.875	**0.048** *	0.417	0.175	0.993

*P-value < 0.05

B: coefficient of regression; AOR: adjusted odds ratio; 95% CI: 95% confidence Interval

## Discussion

Nearly half of the respondents (45.7%) had ever received any form of treatment from any PHC center(s) in the study location. This is similar to the 42.5% reported among 200 household residents in Ogun State, Nigeria (19). The utilization rate in our study is however lower than that of a cross-sectional study among 350 adults conducted in Imesi-Ile, a rural community in Osun State, Nigeria where it was reported that 63.1% of the participants used PHC facilities (16). Generally, PHC utilization rate across the country is lower than what is expected probably due to a lack of trust in the system and ease of access to secondary and tertiary facilities without a referral. The utilization of services in the PHC centers could vary across socio-demographic and socioeconomic profiles. In this study, none of the socio-demographic variables was significantly associated with the utilization of PHC.

The respondents showed high levels of awareness that PHC facilities were noted for providing services such as immunization, mother and child health care, family planning, prenatal care, treatment of common illnesses and injuries, health education, and distribution of vital pharmaceuticals. Despite the high level of awareness of some of the services provided by PHC centers, only a few respondents utilized these services. Mental health and dental and oral health services had the least level of awareness and utilization.

This study found that 79% of the respondents knew there was a PHC facility in their community. This is lower compared to a cross-sectional study of 383 adults aged 15 years and over in Jaba Local Government Area of Kaduna State, Nigeria where almost all (97.9%) of the respondents were aware of the existence of PHC services in their community (20). The level of awareness observed could stem from several awareness campaigns on immunization and other deliverables in the social space by stakeholders. Our findings revealed that those who were aware of a PHC center in their community were found to be 4.28 times more likely to use PHC services than those who did not.

The awareness that PHC centers in the community run all day was found to be a significant factor in predicting the utilization of PHC. Those who were aware of how PHC centers operate were 1.66 times more likely to utilize PHC services than those who did not know. This study found that having the knowledge that members of the community are part of the working staff in the PHC center influences the utilization of PHC. Those who had this awareness were 1.44 times more likely to utilize PHC services compared to those who did not.

Those who perceived that PHC centers lack qualified personnel, especially doctors were less likely to utilize PHC services. The inadequacy of medical personnel is one of the major problems confronting healthcare delivery in Nigeria (21). In 2018, Nigeria had 3.81 doctors for every 10,000 population which puts Nigeria's doctor-population ratio at 1:2,625, which is below WHO's recommendation of one physician to 600 persons. Currently, Nigeria's doctor-patient ratio is 1:4,088 (22). This is because medical doctors (and other medical personnel) are leaving the country because of the higher remuneration abroad and poor pay in Nigeria. In 2021, Nigeria lost nearly 9,000 doctors and other health workers to the UK and other countries within two years. Even though Nigeria produces about 3,000 doctors every year, Nigeria's doctor-population ratio remains low (22). The failure of PHC in Nigeria is partly because of the inadequate number and proportion of the various healthcare workers needed to provide the required services (23). The minimum standard for PHC in Nigeria as recommended by the National Primary Health Care Development Agency is that one PHC center must exist in each political ward. There must be one Medical Officer (if available), one Community Health Officer (CHO), four Nurses/Midwives, three Community Health Extension Workers (CHEW), one Laboratory Technician, six Junior Community Health Extension Workers (JCHEW), one Environmental Officer, one Pharmacy Technician, one Medical Records Officer, two Health Assistants, one General Maintenance staff, and two security personnel in every PHC center (23). Unfortunately, most PHC centers in Nigeria (including Ido-Ekiti) do not have these personnel.

Those who prefer to visit secondary and/or tertiary health institutions were less likely to visit PHC centers in their community. About one-quarter (26.3%) of the participants prefer to visit general/teaching hospitals. Despite being a rural area, Ido-Ekiti has a federal teaching hospital. The hospital, formerly known as Federal Medical Centre, was established in 1998 as a tertiary healthcare facility by the federal government to provide affordable, qualitative, and accessible healthcare. The hospital was later upgraded to a teaching hospital by the federal government after signing a Memorandum of Understanding (MoU) with Afe Babalola University to train the university's medical students. This is likely responsible for the residents of Ido-Ekiti seeking health care at this hospital rather than the PHC centers. The outcomes of this study are similar to a cross-sectional study conducted in Lagos State, where general hospitals were the first point of healthcare for 55% of respondents whenever they became unwell. This is because it is thought that PHC facilities only serve children and pregnant women, lack appropriate medical supplies, and have subpar equipment (24). The study also found that a lack of supplies and medications in the medical facility was the main factor given by most respondents who were dissatisfied with the services offered by PHC clinics. The attitude of medical staff, inadequate trained medical workers, particularly doctors, and PHC centers' restricted services, were reported to have a substantial negative impact and are responsible for the low utilization of PHCs (24).

Fewer than 30% of Nigeria's rural residents have access to contemporary healthcare facilities, despite the fact that the country's rural population is projected to make up 53% of the overall population and be home to 70% of the nation's impoverished (25). This is due to the problem of inadequate coverage for the rural population. Studies have reported that residing in rural areas (26), far distance to health facilities (27–29), and non-availability of experienced staff (30) are significant factors limiting access to and utilization of healthcare services. A limitation of this study is that it is cross-sectional. Hence, a causal association cannot be established. The results obtained from this study are self-reported and are susceptible to exaggeration and recall bias. The government at all levels need to prioritize PHC as this is one of the best ways to deliver complete, universal, and reasonable healthcare services to those living in rural areas. Though the awareness level was high among this study's participants, the perception of the non-availability of medical personnel and easy access to secondary and tertiary health institutions are potential threats to the utilization of PHC facilities.

The local government needs to prioritize PHC in delivering equitable and affordable healthcare services to those living in rural areas. The availability of medical personnel and well-equipped facilities can ameliorate the utilization of PHC services.
